# Comprehensive Clinical Profile and Hemodialysis Outcomes in Patients Attending a Tertiary Care Hospital

**DOI:** 10.7759/cureus.66816

**Published:** 2024-08-13

**Authors:** Nishanth Gejjalagere Chandrashekar, Naresh Vishwanath Iyer Murali, Yogesh S, Awais Ilyas Mohammed, Dharshini Hemachandran, Kailash Narendran, Tejaswee Lohakare

**Affiliations:** 1 Internal Medicine, Our Lady of Fatima University, Valenzuela, PHL; 2 Emergency Medicine, United Lincolnshire Hospitals NHS Trust, Grantham, GBR; 3 Internal Medicine, Bharat Ratna Dr. B.R. Ambedkar Medical College, Bangalore, IND; 4 General Medicine, United Lincolnshire Hospitals NHS Trust, Grantham, GBR; 5 Internal Medicine, Madras Medical College, Rajiv Gandhi Government General Hospital, Chennai, IND; 6 General Medicine, Lincoln County Hospital, Lincoln, GBR; 7 Internal Medicine, Employees' State Insurance Corporation (ESIC) Post Graduate Institute of Medical Science and Research, Chennai, IND; 8 Surgery, East Lancashire Hospitals NHS Trust, Blackburn, GBR; 9 Child Health Nursing, Srimati Radhikabai Meghe Memorial College of Nursing, Datta Meghe Institute of Higher Education and Research, Wardha, IND

**Keywords:** outcome, clinical features, end-stage renal disease (esrd), dialysis, chronic kidney disease (ckd)

## Abstract

Background

Chronic kidney disease (CKD) can lead to serious conditions such as anemia and cardiovascular disease, posing a growing global health challenge. End-stage renal disease (ESRD) requires treatments such as dialysis or kidney transplantation. Despite the widespread impact and rising prevalence of CKD and ESRD, comprehensive data remains limited in India. This study seeks to investigate the clinical, socio-demographic, and etiological profiles of CKD patients undergoing hemodialysis at a tertiary care hospital, with the goal of enhancing understanding and improving patient care.

Methodology

This retrospective cohort study, conducted at a tertiary care center, included 500 CKD patients undergoing hemodialysis, with comprehensive medical records. Data collected covered demographics (age, sex, education, and occupation), CKD etiology, disease duration, hemodialysis duration, viral marker status, blood transfusions, and vascular access details. With continuous variables reported as mean ± standard deviation (SD) and categorical variables as counts (percentages), statistical analysis was carried out using SPSS version 21 (IBM Corp., Armonk, New York, USA). The connections were examined using the Pearson Chi-square test, with P≤0.05 being deemed significant.

Results

The study revealed that hypertension was the primary cause of CKD in 58% of patients, followed by diabetes mellitus in 13%. A significant 93% of patients tested negative for viral markers such as human immunodeficiency virus (HIV), hepatitis C virus (HCV), and hepatitis B surface antigen (HBsAg). Hemodialysis duration varied, with 68% of patients undergoing dialysis for one to five years. Most patients had two (40%) or three (58%) dialysis sessions per week, and 84% had only one arteriovenous (AV) fistula surgery. Blood transfusions were common, with 62% of patients receiving between one and five transfusions. The gender distribution showed more males (372) than females (201), and the majority of patients were aged between 41 and 60 years.

Conclusion

This study highlights the importance of early detection and management of CKD, emphasizing preventive health measures, enhanced diagnostic capabilities, and sufficient resource allocation to reduce the disease burden. It also calls for further research into unknown CKD causes and strategies to improve patient care and outcomes.

## Introduction

Chronic kidney disease (CKD) has become a major global public health issue that impacts people in high-income and low-to-middle-income nations alike. Kidney disease is a degenerative illness that progressively impairs kidney function over time. In CKD, the kidneys, which are vital organs for filtering blood and eliminating waste from the body, suffer damage and inefficiency. This impairment leads to the accumulation of harmful levels of fluids, electrolytes, and metabolic waste products, which can cause various serious health issues, including cardiovascular disease, bone disorders, and anemia [1,2].

The most advanced stage of chronic kidney disease (CKD), known as end-stage renal disease (ESRD), occurs when the kidneys are unable to maintain the vital physiological balance in the body. In this stage, patients often face a significant decline in their quality of life and require ongoing treatment such as dialysis or a kidney transplant to survive. Dialysis involves the use of artificial methods to perform the blood-filtering functions of the kidneys, while kidney transplantation offers a potential cure by replacing the failed kidneys with a healthy donor organ [3].

Kidney disorders are the 12th largest cause of mortality and the 17th leading cause of disability globally, according to the World Health Organization (WHO), which has identified them as a significant global health concern. This recognition underscores the increasing burden of kidney-related ailments across diverse populations. In India, the true extent of CKD and ESRD is challenging to quantify accurately due to the absence of a national renal registry. Without comprehensive data, it is difficult to fully understand the prevalence and impact of these conditions on the population [4,5].

Estimates suggest that the prevalence of ESRD is expected to rise significantly in the future. The population's aging, which is linked to an increased risk of kidney disease, as well as the rising prevalence of chronic illnesses like diabetes mellitus and hypertension, both of which are significant risk factors for the onset and course of CKD, are the main causes of this predicted increase [6].

Given these concerns, the present study was undertaken to provide a detailed examination of the clinical, socio-demographic, and etiological profiles of CKD patients who are undergoing hemodialysis at a tertiary care hospital. By analyzing these factors, the study aims to contribute valuable insights into the characteristics and needs of this patient population, which can inform strategies for improving patient care and addressing the broader public health challenge of CKD and ESRD.

## Materials and methods

Study design and setting

In a tertiary care facility, a retrospective cohort analysis was carried out. The institutional ethics committee gave its permission before the trial could start.

Selection Criteria

The study's selection criteria required the inclusion of case sheets from CKD patients receiving hemodialysis and having complete and pertinent medical records. To be included, case sheets needed to provide complete details about the patient’s demographic profile, clinical history, treatment regimens, and laboratory results. In contrast, case sheets were excluded if they contained incomplete or insufficient information, such as missing clinical data or treatment details, which would compromise the accuracy and reliability of the analysis.

Data sources and variables

Case sheets for a total of 500 patients were retrieved and reviewed in accordance with the established inclusion criteria. From these records, a comprehensive set of data was extracted to ensure a thorough understanding of the patient population. The socio-demographic profiles collected included age (in years), sex, educational status, and occupation of each patient. This information provided insights into the demographic characteristics and socioeconomic backgrounds of the individuals undergoing treatment.

Additionally, clinical data was meticulously gathered from the case sheets. This data encompassed the etiology of CKD as specified by the treating physician, which helped identify the underlying causes contributing to each patient's condition. The duration of CKD, as well as the length of time each patient had been undergoing hemodialysis, was recorded to assess the progression and treatment history of the disease.

Further clinical details included the status of viral markers, which are critical for understanding potential complications or co-infections associated with CKD. The number of blood transfusions each patient had received was noted, providing insight into the severity of anemia or other related conditions. Information on vascular access, including the type and number of failed arteriovenous (AV) fistulas, was also extracted. This data is essential for evaluating the adequacy of vascular access and the challenges faced in managing hemodialysis.

Statistical analysis

SPSS version 21 (IBM Corp., Armonk, New York, USA) was used for the analysis after the data was imported into Microsoft Excel (Microsoft Corporation, Redmond, Washington, USA). Continuous variables were reported as mean ± standard deviation (SD) in the descriptive analysis, whereas categorical variables were expressed as count (percentages). The correlation between categorical variables was investigated using the non-parametric Pearson Chi-square test. P-values less than 0.05 were regarded as statistically significant.

## Results

Table 1 shows the clinical characteristics of the patients. The primary cause of CKD includes hypertension (HTN) in 290 patients (58%), diabetes mellitus (DM) in 65 patients (13%), polycystic kidney disease (PCKD) in 45 patients (9%), acute renal failure (ARF) in 35 patients (7%), uropathy in 12 patients (2.4%), and an unknown cause in 53 patients (10.6%). Regarding viral marker status, 465 patients (93%) were negative for human immunodeficiency virus (HIV), hepatitis C virus (HCV), and hepatitis B surface antigen (HBsAg), while 35 patients (7%) were positive. The duration of hemodialysis was less than one year in 65 patients (13%), between one and five years in 340 patients (68%), between six and ten years in 75 patients (15%), and more than eleven years in 20 patients (4%). The frequency of hemodialysis sessions per week was two for 200 patients (40%), three for 290 patients (58%), and four for 10 patients (2%). The number of AV fistula surgeries was one for 420 patients (84%), two for 55 patients (11%), and three or more for 25 patients (5%). The number of blood transfusions to date was none for 130 patients (26%), between one and five for 310 patients (62%), between six and ten for 50 patients (10%), and eleven or more for 10 patients (2%).

**Table 1:** Clinical characteristics of the patients (n=500). HIV: Human immunodeficiency virus, HCV: hepatitis C virus, HBsAg: hepatitis B surface antigen, AV: arteriovenous.

Clinical profile	Frequency	Percentage (%)
Primary cause of CKD		
Hypertension (HTN)	290	58
Diabetes mellitus (DM)	65	13
Polycystic kidney disease (PCKD)	45	9
Acute renal failure (ARF)	35	7
Uropathy	12	2.4
Unknown	53	10.6
Viral marker status (HIV, HCV, HBsAg)		
Negative	465	93
Positive	35	7
Duration of hemodialysis (years)		
<1	65	13
1-5	340	68
6-10	75	15
≥11	20	4
Frequency of hemodialysis sessions/week		
2	200	40
3	290	58
4	10	2
Number of AV fistula surgeries		
1	420	84
2	55	11
≥3	25	5
Number of blood transfusions to date		
None	130	26
1-5	310	62
6-10	50	10
≥11	10	2

Table 2 presents the demographic attributes of CKD patients categorized by the etiology of their condition. The age distribution across different etiologies shows that in patients with ARF, three were ≤20 years old, 12 were between 21 and 40 years, 14 were between 41 and 60 years, and 19 were ≥61 years. For DM patients, two were ≤20 years, 27 were between 21 and 40 years, 32 were between 41 and 60 years, and 17 were ≥61 years. Among HTN patients, 12 were ≤20 years, 115 were between 21 and 40 years, 165 were between 41 and 60 years, and 33 were ≥61 years. PCKD patients had one patient ≤20 years, 22 patients between 21 and 40 years, 29 patients between 41 and 60 years, and five patients ≥61 years. Uropathy patients had no one ≤20 years, six patients between 21 and 40 years, 11 patients between 41 and 60 years, and two patients ≥61 years. Patients with unknown etiology had two patients ≤20 years, 26 between 21 and 40 years, 30 between 41 and 60 years, and 7 ≥61 years. Educational attainment varied, with 110 patients being college graduates, 148 having a high school diploma, 297 with some college education, and 53 with middle school education. Job classification showed 206 professionals, 157 manual laborers, and 250 not employed. Gender distribution was 201 females and 372 males.

**Table 2:** Demographic attributes of CKD patients by etiology (n=500). p-value < 0.05 is considered to be statistically significant. CKD: chronic kidney disease.

Attributes	Acute renal failure (ARF)	Diabetes mellitus (DM)	Hypertension (HTN)	Polycystic kidney disease (PCKD)	Uropathy	Unknown	Total	P-value
Age category (years)								
≤20	3	2	12	1	0	2	20	0.620
21-40	12	27	115	22	6	26	208	
41-60	14	32	165	29	11	30	289	
≥61	19	17	33	5	2	7	70	
Educational attainment								
College graduate	3	18	65	4	1	9	110	0.485
High school diploma	14	18	80	16	2	18	148	
Some college	19	40	165	28	13	32	297	
Middle school	3	6	25	6	2	10	53	
Job classification								
Professional	15	24	125	18	18	6	206	0.095
Manual laborer	15	16	80	20	16	10	157	
Not employed	15	38	135	22	33	7	250	
Gender								
Female	10	28	115	16	5	27	201	0.380
Male	30	47	210	36	15	34	372	

Table 3 details the profile of patients based on the duration of their CKD. Age-wise, 20 patients were ≤20 years old, with 15 having CKD for 0-5 years, three for 6-10 years, one for 11-15 years, and one for ≥16 years. Among patients aged 21-40 years, 115 had CKD for 0-5 years, 45 for 6-10 years, 10 for 11-15 years, and five for ≥16 years. Patients aged 41-60 years included 188 with CKD for 0-5 years, 46 for 6-10 years, 10 for 11-15 years, and six for ≥16 years. In the ≥61 years category, 42 had CKD for 0-5 years, 13 for 6-10 years, three for 11-15 years, and two for ≥16 years. Dialysis frequency per week showed 205 patients undergoing two sessions, 290 undergoing three sessions, and five undergoing four or more sessions. The number of AV fistulas showed 402 patients with one fistula, 60 with two, and 25 with three or more.

**Table 3:** Profile of patients with duration of CKD (n=500). p-value < 0.05 is considered to be statistically significant. CKD: chronic kidney disease.

Characteristics	0-5 years	6-10 years	11-15 years	≥16 years	Total	P-value
Age category (years)						
≤20	15	3	1	1	20	0.982
21-40	115	45	10	5	175	
41-60	188	46	10	6	250	
≥61	42	13	3	2	60	
Dialysis frequency/week						
Two sessions	147	44	9	5	205	0.834
Three Sessions	205	63	15	7	290	
≥4 Sessions	2	1	1	1	5	
Number of AV fistulas (AVF)						
1	302	80	10	10	402	0.002
2	35	23	1	1	60	
≥3	11	2	11	1	25	

Table 4 describes the characteristics of patients undergoing maintenance hemodialysis (MHD). The number of AVF failures was none in 317 patients (40 ≤1 year, 220 for two to five years, 55 for six to 10 years, and two for ≥11 years), one in 171 patients (22 ≤1 year, 116 for two to five years, 22 for six to 10 years, and 11 for ≥11 years), and two in 12 patients (all in the two to five years category), with a P-value of 0.006. Blood transfusion incidents showed 131 patients with none (29 ≤1 year, 75 for two to five years, 25 for six to 10 years, and two for ≥11 years), 305 with one to five transfusions (33 ≤1 year, 250 for two to five years, 11 for six to 10 years, and 11 for ≥11 years), 54 with six to 10 transfusions (all in the six to 10 years category), and 13 with 11 or more transfusions (all in the two to five years category), with a P-value of 0.002. Viral marker status was negative in 457 patients (57 ≤1 year, 320 for two to five years, 68 for six to 10 years, and 12 for ≥11 years) and positive in 38 patients (five for ≤1 year, 22 for two to five years, 10 for six to 10 years, and one for ≥11 years), with a P-value of 0.830.

**Table 4:** Characteristics of patients undergoing maintenance hemodialysis (MHD) (n=500). p-value < 0.05 is considered to be statistically significant. AVF: AV fistulas.

Category	≤1 year	2-5 years	6-10 years	≥11 years	Total	P-value
Number of AVF failures						
None	40	220	55	2	317	0.006
One	22	116	22	11	171	
Two	0	12	0	0	12	
Blood transfusion Incidents						
None	29	75	25	2	131	0.002
1-5	33	250	11	11	305	
6-10	0	12	42	0	54	
11 or more	0	13	0	0	13	
Viral marker status						
Negative	57	320	68	12	457	0.830
Positive	5	22	10	1	38	

## Discussion

Globally, one of the main causes of death and morbidity is chronic kidney disease or CKD. Conservatively treated early-stage CKD eventually progresses to ESRD, necessitating renal replacement therapies like dialysis or transplantation. The present study sheds light on the clinical and demographic characteristics of patients with chronic kidney disease receiving hemodialysis at a tertiary care facility.

In the current study, the medical records of 500 patients were analyzed. There were 128 female patients (26%) and 372 male patients (74%), making the male-to-female ratio of these individuals 2.9:1. According to several studies on the subject, male gender has been identified as a significant risk factor for the development of CKD [7-9].

The current study's mean age of presentation was 45 years, which is consistent with CKD's chronic character. Other research done in other regions of India has discovered similar results [10,11]. Global data, on the other hand, demonstrates that the prevalence of CKD is higher in those 65 years of age or older (38%) than in those 45-64 years (13%) or 18-44 years (7%), respectively [2]. In India and Pakistan, the average age of patients in need of renal replacement therapy (RRT) is lower than in the industrialized countries. Several things appear to be at play, such as the lack of access to quality healthcare, which causes delays in diagnosis and misses opportunities to implement prompt preventative interventions [12].

The most common associated factor in the current study was hypertension (HTN), affecting 290 patients (58%), followed by DM in 65 patients (13%). This result is in line with research by Ruggenenti et al. [13] and Hill et al. [3], which found that the most frequent cause of CKD was hypertension. Nonetheless, recent research conducted in various regions of India suggests that the most frequent cause of ESRD is diabetic nephropathy [7,10,11,14]. The disparity in the etiology of CKD might be brought about by changes in the incidence of these non-communicable illnesses, variances in the racial and ethnic composition of the study population, or both.

A significant percentage of CKD cases in the current study had an unknown etiology (10.6%), consistent with the etiological diagnosis reported by Jha [12]. The unknown etiology needs to be explored further to adopt more preventive and curative modalities for early intervention. Notably, glomerulonephritis was found to be the most common cause of CKD in a study by Dharan et al. [15], while obstructive uropathy was significant in geriatric patients, according to another study [14]. In the current study, uropathy accounted for 2.4% of cases.

Regarding vascular access, the current study found that 15% of patients required multiple arteriovenous fistulas (AVFs). The reasons for multiple AVF placements could include failure due to technician error, infections due to improper hygiene, hypotension during hemodialysis, or poor maintenance of the AVF by patients themselves. The current findings also revealed that 62% of patients had a history of one to five blood transfusions, while 26% had not required any transfusions. The need for multiple transfusions may be attributed to severe anemia resulting from erythropoietin deficiency, a common complication of CKD.

In the context of viral infections, 7% of patients were positive for HIV, HCV, or HBsAg. This is similar to the findings from a study in Tripura, which reported hepatitis B and C positivity rates of 5.5% and 10.2%, respectively [16]. The increased risk of viral infections can be linked to multiple hospital visits for hemodialysis sessions and blood transfusions, which elevate the chances of acquiring infections during these procedures. The overall results of the current study align closely with those observed in the study conducted by Kumar et al. [17]. This concordance suggests a consistency in findings across different research settings, reinforcing the reliability and validity of the data regarding the clinical and demographic characteristics of CKD patients. Such alignment also underscores the prevalence of common etiological factors and treatment outcomes in CKD patients, highlighting similar patterns and trends observed in both studies. Yagi et al., in their study, also stressed the burden caused by CKD and how it hampers the daily lifestyle and living of patients [18]. 

Traditionally, diabetes mellitus, cardiovascular disease (CVD), and hypertension have been the main targets of health initiatives aimed at preventing chronic illnesses. However, there is an urgent need to prioritize early diagnosis and patient referral to nephrology departments due to the increasing frequency of CKD advancing to ESRD and the substantial cost burden of renal replacement therapy (RRT). Reducing the burden of CKD requires additional funding for the treatment of CKD/ESRD patients and efficient planning for preventative health measures. In addition to improving patient outcomes, early intervention, thorough treatment, and routine monitoring can lower the overall costs of care related to CKD.

Limitations of the study

The retrospective design of the current study is one of its weaknesses; it may create selection bias and make it more difficult to prove causation. Furthermore, the study was carried out at a single tertiary care facility, which might not be an accurate representation of the entire community of patients with CKD. Reliance on medical records can sometimes lead to inadequate or erroneous data, especially when it comes to the history of viral infections and blood transfusions, as well as the etiology of chronic kidney disease. Lastly, the study's cross-sectional design makes it impossible to evaluate long-term results and changes across time.

## Conclusions

This study underscores the need for improved early detection and management of CKD, particularly in regions with high disease prevalence and varying etiological factors. Emphasis on preventive health measures, enhanced diagnostic capabilities, and adequate resource allocation for CKD and ESRD patients are critical steps in reducing the overall burden of this disease. Future research should continue to explore unknown causes of CKD and evaluate strategies to optimize patient care and outcomes.
